# Use of Blood Smears and Dried Blood Spots for Polymerase Chain Reaction–Based Detection and Quantification of Bacterial Infection and *Plasmodium falciparum* in Severely Ill Febrile African Children

**DOI:** 10.4269/ajtmh.15-0532

**Published:** 2016-02-03

**Authors:** Benchawan Wihokhoen, Arjen M. Dondorp, Paul Turner, Charles J. Woodrow, Mallika Imwong

**Affiliations:** Department of Molecular Tropical Medicine and Genetics, Faculty of Tropical Medicine, Mahidol University, Bangkok, Thailand; Mahidol-Oxford Tropical Medicine Research Unit, Faculty of Tropical Medicine, Mahidol University, Bangkok, Thailand; Centre for Tropical Medicine, Churchill Hospital, University of Oxford, Oxford, United Kingdom; Cambodia-Oxford Medical Research Unit, Angkor Hospital for Children, Siem Reap, Cambodia

## Abstract

Molecular approaches offer a means of testing archived samples stored as dried blood spots in settings where standard blood cultures are not possible. Peripheral blood films are one suggested source of material, although the sensitivity of this approach has not been well defined. Thin blood smears and dried blood spots from a severe pediatric malaria study were assessed using specific polymerase chain reaction (PCR) primers to detect non-typhoidal *Salmonella* (NTS; *MisL* gene), *Streptococcus pneumoniae* (*lytA*), and *Plasmodium falciparum* (18S rRNA). Of 16 cases of NTS and *S. pneumoniae* confirmed on blood culture, none were positive by PCR using DNA extracts from blood films or dried blood spots. In contrast, four of 36 dried blood spots and two of 178 plasma samples were PCR positive for *S. pneumoniae*, despite negative bacterial blood cultures, suggesting false positives. Quantitative assessment revealed that the effective concentration of *P. falciparum* DNA in blood films was three log orders of magnitude lower than for dried blood spots. The *P. falciparum kelch13* gene could not be amplified from blood films. These findings question the value of blood PCR-based approaches for detection of NTS and *S. pneumoniae*, and show that stored blood films are an inefficient method of studying *P. falciparum*.

## Introduction

Non-malarial infections form an increasing proportion of acute febrile illnesses in African children^1^ and are a major cause of death among children in rural areas.^2^ A malaria blood film or rapid diagnostic test is often the only diagnostic available, but the presence of parasites does not rule out other serious conditions.^3^ Approximately, a quarter of patients thought to have cerebral malaria in fact have other diagnoses on postmortem.^4^ There is also emerging evidence that patients with severe malaria have a higher chance of concomitant invasive bacterial infection.^5^,^6^

An understanding of the range of non-malarial infections in these settings is critical for the development of improved management algorithms and novel diagnostic tests for non-malarial illnesses. Presumptive treatment with antibiotics according to simple clinical criteria is included in the integrated management of childhood illness algorithms, but it is likely that antibiotics are still poorly targeted; for example, in a large study of febrile Tanzanian children, less than 13% of children with acute respiratory infection required treatment with antibiotics.^1^ Detailed research surveys using a range of diagnostic methods can provide a picture of the range of causes of febrile illness in a particular setting.^1^,^7^ However, the majority of locations currently have no access to diagnostic tests for bacterial and other causes of non-malarial febrile illness.

Diagnosis of invasive bacterial infection using molecular methods has been studied and implemented in well-resourced settings to supplement standard bacterial culture methods.^8^ Polymerase chain reaction (PCR) of whole blood samples can identify organisms even when there has been antibiotic pretreatment, and direct amplification of patient samples (without prior culture) potentially offers a result time of a few hours. Commercial kits are available involving direct multiplex real-time PCR on blood samples based on conserved rRNA-based sequences, although the accuracy of such approaches appears to vary widely across studies.^9^ Use of capillary sequencing of products can potentially add specificity to this approach. In general, antibiotic susceptibility results cannot generally be obtained by molecular methods.

In this work, we examine the use of dried blood spots, malaria films, and plasma samples for PCR-based detection of two common invasive bacterial pathogens (*Streptococcus pneumoniae* and non-typhoidal *Salmonella* [NTS]), as well as *Plasmodium falciparum*, in the context of the artesunate versus quinine severe falciparum malaria trial in African children (African Quinine Artesunate Malaria Trial [AQUAMAT]). We aimed to examine the accuracy of PCR using archived material for detection of bacterial infection and quantification of *P. falciparum*, using blood cultures and microscopy, respectively, as gold standards.

## Materials and Methods

### Clinical samples and data.

All samples in this study were obtained from children enrolled in Muheza, Tanzania, in 2007 in AQUAMAT.^5^,^10^ The blood culture–positive set consisted of samples from 26 patients, including 12 with NTS species and two with *S. pneumoniae* as well as cases positive for *Staphylococcus aureus* (four), *Burkhoderia cepacia* (two), *Stenotrophomonas maltophilia* (two), *Pseudomonas stutzeri* (one), and *Klebsiella pneumoniae* (one). DNA extracts were obtained from both dried blood spots and blood smears from the 16 cases of NTS and *S. pneumoniae* to assess the presence of the relevant organisms by PCR and for *P. falciparum* molecular quantitation; extracts from dried blood spots were also obtained for the 10 other blood culture–positive cases and entered the same PCR reactions. Additional sets of blood culture–negative samples from the Muheza AQUAMAT site were used as templates for testing the specificity of NTS- and *S. pneumoniae*-specific PCRs. To enrich for possible bacteremic cases,^5^ 36 dried blood spot extracts from culture-negative patients with extremes of plasma *P. falciparum* histidine-rich protein 2 (PfHRP2) described previously^11^ were tested (either less than 46 ng/mL or greater than 11,000 ng/mL baseline plasma PfHRP2). In addition, all available plasma samples from the first 200 culture-negative patients enrolled in the Muheza site (178 samples) were tested. There was no significant difference in parasitemia among these various sets of samples.

### Sample preparation.

Each Giemsa-stained thin blood film, estimated to contain 5 μL of blood, was scraped off using a clean scalpel blade and collected in a microfuge tube. Dried blood spots were punched out to yield an estimated 20 μL blood and placed in a microfuge tube. DNA extraction was undertaken using the QIAamp DNA Mini Kit (Qiagen, Valencia, CA) with a final suspension volume of 50 μL (blood films) and 100 μL (dried blood spots). For each PCR reaction, 2 μL DNA extract was used; the same volume of plasma was used directly in the PCR reaction without purification.

### Real-time PCR for *S. pneumoniae* and NTS spp.

Real-time PCR for *S. pneumoniae* and NTS spp. was performed using previously described primers.^12^ For *S. pneumoniae*, primers amplified the autolysin-encoding gene *lytA*, a cell wall-degrading enzyme located in the cell envelope of *S. pneumoniae*^13^: forward primer 5′-TTGGGAACGGTTGCATCATG-3′, reverse primer 5′-TCGTGCGTTTTAATTCCAGCT-3′. For NTS spp., primers amplified the autotransporter protein *misL* encoded by *Salmonella* pathogenicity island 3^14^: forward primer 5′-GACGTTGATAGTCTGCCATCCAC-3′, reverse primer 5′-CAATGCCGCCAGTCTCCGTGC-3′. Bacterial PCRs were performed using the StepOnePlus real-time PCR system (Applied Biosystems, Foster City, CA) with a thermal profile of 2 minutes at 98°C, 45 cycles of 5 seconds at 98°C, and 30 seconds at 60°C with dissociation 15 seconds at 95°C, 1 minute at 60°C (+3°C), and 15 seconds at 95°C. Each reaction was performed in a volume of 20 μL mixing SsoFast EvaGreen (BioRad, Hercules, CA) with 0.25 μM of each primer and 2 μL DNA extract or plasma.

### Standards.

Bacterial DNA-positive and -negative control standards were from the Biodefense and Emerging Infections Research Resources Repository (BEI Resources) on website http://www.beiresources.org/ (Table 1). To confirm positive samples in the *S. pneumoniae* real-time PCR, products were sequenced commercially and aligned with reference *lytA* sequence GenBank AJ243414.1.

### Real-time PCR for *Plasmodium* spp. and *P. falciparum*.

A species-specific assay was used to confirm *P. falciparum* infection, using established primers, probe, and conditions^15^; forward primer was 5′-CTTTTGAGAGGTTTTGTTACTTTGAGTAA-3′, reverse primer 5′-TATTCCATGCTGTAGTATTCAAACACAA-3′, and probe sequence 5′-TGTTCATAACAGACGGGTAGTCATGATTGAGTTCA-3′ labeled with 5′ FAM and 3′ TAMRA as reporter and quencher, respectively. A real-time generic *Plasmodium* spp. assay was then used to quantify gene copies of 18S rDNA using established primers, probe, and conditions^16^; forward primer was 5′-GCTCTTTCTTGATTTCTTGGATG-3′, reverse primer 5′-AGCAGGTTAAGATCTCGTTCG-3′, and probe sequence 5′-ATGGCCGTTTTTAGTTCGTG-3′.

Amplification and real-time measurements were performed in the 5-plex Rotorgene Q (Qiagen) with the following thermal profile for quantitative PCR (qPCR): 10 minutes at 95°C, 50 cycles of 15 seconds at 95°C, and 1 minute at 60°C. For the reaction, 2 μL template was added to 8 μL reaction master mix containing 1× QuantiTect Multiplex PCR Master Mix NoROX (Qiagen), 0.4 μM of each primer, and 0.2 μM probe.

Relevant primers derived from the *kelch13* gene sequence were used to amplify the full *kelch13* open reading frame using a nested PCR protocol.^17^

## Results

### Standards.

Analyses of individual melting curves for *S. pneumoniae* and *Salmonella* spp. amplification showed melting temperatures of approximately 83°C and 87°C, respectively. The limit of detection for the *S. pneumoniae*-specific assay was 2.8 × 10^−6^ μg/mL, and 1.62 × 10^−5^ μg/mL for the *Salmonella* spp.-specific assay, based on BEI templates NR4218 and HM-145D, respectively. Calculations based on established estimated molecular weights for bacterial genomes^18^ confirmed that this limit of detection translated to less than 100 copies/mL, indicating that both PCR reactions were highly efficient.

### Bacterial PCR.

None of the 240 samples studied were positive for NTS by PCR, whether undertaken on extracts from blood smear, dried blood spot, or directly from plasma (Table 2). This included 14 patients with positive blood cultures.

The two *S. pneumoniae* culture–positive cases were negative by PCR from dried blood spot and blood smear extracts and plasma (Table 2). However, other cases that were culture negative were positive by PCR, either using dried blood spots (four cases) or plasma (two cases) as template. In these samples, melting curve analysis showed values close to control values (approximately 83°C) and threshold cycles (CT) consistent with significant amounts of DNA (between 34.2 and 37.6 cycles). PCR products were also confirmed to be genuine products of *S. pneumoniae* by sequencing (more than 90% identity when aligned with the *S. pneumoniae lytA* reference sequence).

### *P. falciparum* quantitative PCR.

*P. falciparum* genomic quantitation based on dried blood spot extracts was undertaken for the 26 blood culture–positive samples and quantitation based on blood smear extract for the subset of 16 NTS- and *S. pneumoniae*-positive cases. Gene copy numbers of 18S rDNA derived from PCR of dried blood spots were broadly in line with predictions from determining organism load by microscopy; mean ± standard deviation [SD] log parasitemia/mL was 6.988 ± 1.253 compared with gene copies/mL of 6.205 ± 1.005. In contrast, genomic quantitation based on blood smears taken at the same time was substantially lower than microscopy; mean copies (18S rDNA)/mL = 2.695 ± 2.265, indicating a drop in detection efficiency of around three log units (*P* < 0.0001 in paired analysis of dried blood spot versus smear).

**Figure 1.**
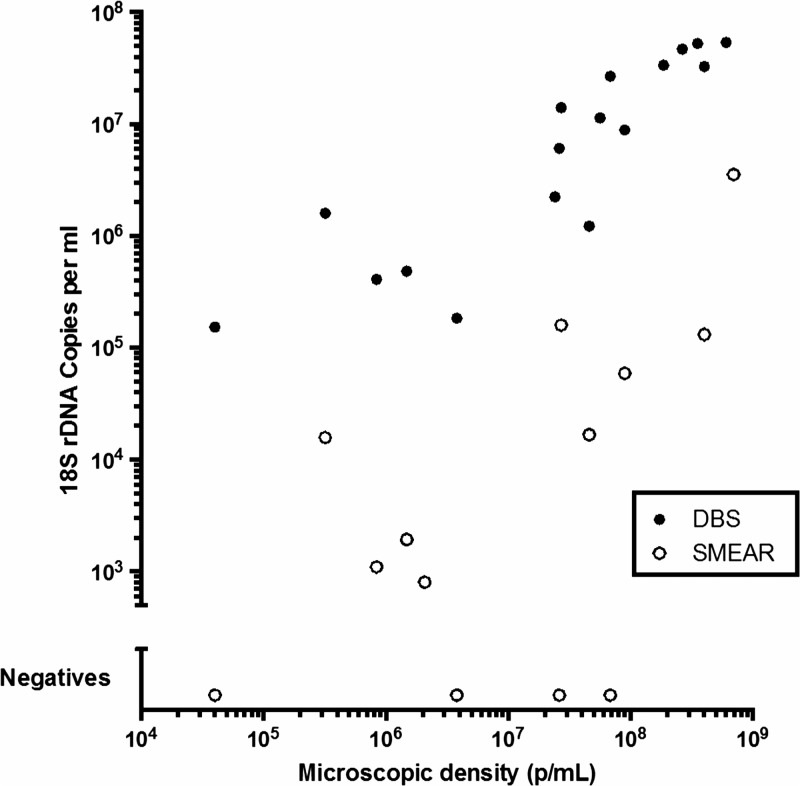
Relationship between parasitemia and 18S rDNA copies/mL in DNA extracts derived from dried blood spots (DBS) and thin blood smears (SMEAR).

There was a statistically significant correlation between measured parasitemia and gene quantity using the dried blood spot (Spearman *r* = 0.909, *P* < 0.0001), and a trend toward a correlation between measured parasitemia and gene quantity using blood smears (Spearman *r* = 0.546, *P* = 0.0566; Figure 1

). In four samples, the blood smear extract was negative by PCR for *P. falciparum*. None of the three segments of the *kelch13* gene (total length 2,181 base pairs [bp]) could be amplified from any of the blood smear extracts.

## Discussion

Molecular detection of malaria and bacterial DNA using dried blood spots, a form of blood storage originally used for detection of genetic conditions, potentially allows assessment of pathogens in areas where cold chain storage is not available. In the case of malaria, this approach has already proven valuable, with qualitative assessment of malaria species and biological markers such as drug resistance polymorphisms now widely undertaken using dried blood spots^19^ or rapid diagnostic tests.^20^ The results we report here are consistent with this experience, with a clear correlation between measured parasitemia and the number of *P. falciparum* gene copies measured in DNA extracts derived from dried blood spots. The dried blood spots used in this case were stored for approximately 5 years with silica gel before DNA extraction, but there was apparently not a substantial loss in terms of gene copies, a well-recognized problem seen with long-term storage of dried blood spots.^21^,^22^ This may depend on the length of the PCR product in question; quantitative studies undertaken here were based on a relatively short product (98 bp in length) from 18S rDNA. Testing across a range of sequence lengths would allow this question to be examined precisely.

Several reports have also described the extraction of DNA from peripheral blood films used for malaria diagnosis, with successful detection of several malaria species.^23^–^28^ This technique is particularly suited to historical samples stored routinely over prolonged periods collected in the course of previous studies.^24^ DNA obtained from thick blood smears showed substantially lower sensitivity than dried blood spots when compared directly.^28^ Our data, using thin blood films, are consistent with these reports, and indicate that blood smears can be useful sources of parasite material, although effective template concentrations appear to be around three log orders of magnitude lower than for dried blood spots, when comparing films and spots made at the same time. Possible reasons for this reduction in sensitivity include the differing storage conditions, loss of parasite material during removal of material from the slide, and inhibitors of DNA extraction and/or PCR because of the chemical properties of Giemsa stain.

There is somewhat less experience with the use of dried blood spots and blood films for bacterial detection. In our case, both dried blood spots and malaria films were clearly insensitive methods for diagnosis of NTS infections, since all 14 blood culture–positive cases were negative by PCR of stored blood material. The relatively low volume of whole blood entering PCR from dried blood spots or smears (less than 1 μL compared with 1–3 mL for blood culture) is likely to be a major factor, given that organism burdens are generally known to be low (median 1 colony-forming unit/mL^29^).

PCR-based detection of *S. pneumoniae* in blood has been the subject of a large number of studies because culture-based diagnosis can be difficult.^8^ The small number of *S. pneumoniae* blood culture–positive cases studied here precludes formal calculation of accuracy for PCR-based diagnosis of *S. pneumoniae*. Several blood culture–negative cases were PCR-positive on dried blood spot samples or plasma samples; could these have been PCR-positive, culture-negative cases of invasive bacterial infection? Although such an interpretation has been suggested by a number of studies,^30^,^31^ there is evidence that *S. pneumoniae* PCR positivity in blood can be found in a significant proportion of healthy children.^30^,^32^,^33^ A cautious approach is probably therefore still justified in interpreting these data.

Our experience differs from a recent PCR-based study based on malaria blood films in which several bacterial species (including *S. pneumoniae*, *Salmonella* spp., and *Borrelia* spp.) were detected by PCR.^12^ Our data comparing blood films with dried blood spots for *P. falciparum* quantitation indicate that the quantity of DNA that can be derived from blood films is substantially lower than that from dried blood spots. The sensitivity of blood films for diagnosis of common invasive bacterial infections is therefore likely to be very low.

## Figures and Tables

**Table 1:** Control DNA templates

BEI codes	Species	Concentrations (μg/mL)
HM-145D	*Streptococcus pneumoniae*	28
HM-248D	*Streptococcus* sp.	27
HM-262D	*Streptococcus mitis*	29
NR-4218	*Salmonella typhimurium*	162
NR-4592	*Salmonella muenchen*	129
NR-4614	*S. typhimurium*	99

**Table 2:** Main results

Blood culture result		Origin of PCR template	PCR result	
Organism	*n*		*Streptococcus pneumoniae*	NTS
NTS	14*	SMEAR, DBS	0	0
*S. pneumoniae*	2	SMEAR, DBS, plasma	0	0
Other organisms	10	DBS	0	0
Negative	36	DBS^11^	4 (11.1%)	0
	178	Plasma^5^	2 (1.1%)	0

DBS = dried blood spots; NTS = non-typhoidal *Salmonella*; PCR = polymerase chain reaction; SMEAR = thin blood smears.

* Dried blood spots were available only in 12 of the 14 NTS-positive cases.
